# Microbial Burden Estimation of Food Items, Built Environments, and the International Space Station Using Film Media

**DOI:** 10.3390/microorganisms10091714

**Published:** 2022-08-25

**Authors:** Anna C. Simpson, Takeo Suzuki, Daniel R. Miller, Kasthuri Venkateswaran

**Affiliations:** 1 Jet Propulsion Laboratory, California Institute of Technology, 4800 Oak Grove Drive, Pasadena, CA 91109, USA; 2 Research & Development Division, Kikkoman Corporation, Noda 278-0037, Japan

**Keywords:** CFU, International Space Station, environmental microbiome, agar media, film media, Easy Plate, Petrifilm

## Abstract

The use of film media involves considerably less preparation, waste, and incubator space than conventional agar-media-based assays and has proven in past studies to provide counts of cultivable microbes similar to those of traditional agar media. Film media also have the advantage of allowing sample volumes similar to those used in pour plates and, therefore, are well-suited for cultivable microbial counts in extremely low-biomass environments such as clean rooms or space habitats, particularly where the subsequent isolation of colonies is necessary. As the preparation of film media plates relies on water cohesion/adhesion rather than manual spreading, they may have future applications in low- or microgravity settings. In this study, cultivable microbial count performance was compared between agar media and film media in three kinds of samples: food items, surfaces in built environments on Earth (homes), and on the environmental surfaces of the International Space Station (ISS). Easy Plates (Kikkoman Corporation) and Petrifilm (3M) were compared with traditional agar plating for food and home surfaces, while only Easy Plates were compared with agar for ISS samples. For both food items and built environments on Earth, both types of film media performed comparably to agar media for bacterial counts, with R^2^ values of 0.94–0.96. Fungal counts for built-environment samples had a lower correlation between film and agar counts, with R^2^ values of 0.72–0.73. Samples from the ISS, which ranged from below detection to 10^3^ CFU per 100 cm^2^, had R^2^ values of 0.80 for bacterial counts and 0.73 for fungal counts, partially due to multiple samples recording below the detection limit for agar or too numerous to count, and the growth of fungal species on R2A medium. The species compositions of isolates picked from agar vs. film media plates were similar; however, further phylogenetic analysis is needed to confirm the differential microbial diversity composition. Overall, film media such as Easy Plates and Petrifilm are viable alternatives to agar plates for low-biomass built environments as well as for food samples, and the two brands tested in this study performed equally well.

## 1. Introduction

Counts of cultivable bacteria and fungi from food items, water samples, and surfaces are a traditional mainstay for public health standards and assessment, as well as the core method that NASA uses to ensure that any craft or robot launched into space meets planetary protection regulations [1]. Preparing, plating, incubating, and counting samples using traditional agar plates, however, generates a great deal of plastic and organic waste and can be prohibitive in terms of the time and space required. Obtaining cultivable microbial counts from low-biomass arenas, including pure water samples, hospitals, clean rooms, and space habitats such as the International Space Station (ISS), presents additional challenges. The samples from these environments are often below detection limits when using standard plating methodology and dilutions [2,3,4], requiring larger numbers and volumes of samples to characterize microbial bioburden [5]. During a survey of multiple NASA clean rooms, for example, more than 30% of samples were below the detection limit for measuring the number of cultivable microbes [6]. In addition, the whole-genome sequencing of isolated fungi and bacteria from cultivable approaches is often necessary for further phenotypic and genomic characterization, which can be used to understand the functional and physiological traits of cultivable microorganisms. For example, isolates from hospitals and human habitats are often assessed for changes or increases in virulence and antibiotic resistance, e.g., [7,8,9]. Similarly, the NASA community is in need of understanding the yet-to-be cultured microorganisms for their possible survival or proliferation in extraterrestrial conditions [10,11,12,13]. Therefore, any improvements or changes in plating methods must not only produce cultivable microbial counts comparable to traditional methods but must also not change the ratios or types of the isolated species.

For the last 50 years, miniaturized stackable media called “film” media have been available as alternatives to traditional agar [14]. Petrifilm was developed by 3M in the 1980s as an alternative to agar spread and pour plates. It consists of a thin wafer of nutrient/gelling agent between two plastic films. Petrifilm was designed to plate 1 mL of a sample on a surface with the approximate size and volume of a playing card, using dyes such as tetrazolium to indicate colony growth [15]. This greatly reduces both the volume of incubator space needed to grow a large number of samples and the time and materials needed to prepare agar plates. According to 3M, Petrifilm media “take up 85% less space, reduce waste 66%, greenhouse gas emissions by 75%, and water waste by 79%” compared with agar plates [15]. Recently, Kikkoman/Dai Nippon Printing (DNP) has developed another brand of film media, Easy Plates, which are similar to Petrifilm in many respects but which have the advantage of a hydrophobic resin ring surrounding the medium which negates the use of a spreader tool, reducing preparation time even further. In addition, the Easy Plate design purportedly increases the ease of counting by its reduced liquefaction of the gel compared with Petrifilm [16].

Previous reports on Petrifilm media found that they produced statistically similar results for cultivable microbial counts to those of agar plate methods for food and environmental safety testing, including aerobic bacteria in raw milk [17], poultry [18], fungal counts on dairy products [19], food items spiked with a variety of common molds [20], and aerobic or coliform bacteria in wastewater [21]. More recent studies showed that film media obtained equivalent counts to agar for soil samples inoculated with varying concentrations of *E. coli* [22], steel and porcelain surfaces inoculated with ubiquitous built-environment bacteria [23], biofilm samples using drop plating [24], and bacterial counts on a variety of food items [25]. There has also been extensive testing of film media in the dairy industry which is leading to the adoption of Petrifilm as a standard method for the enumeration of aerobic and coliform bacteria [26,27,28,29]. Petrifilm is now becoming a widely used method of bacterial and fungal cultivable microbial counting and isolation in place of agar media [29,30].

Most studies testing film vs. agar media performance for cultivable microbial counts have focused on high-bioburden environments and samples such as dairy products and food, or samples/surfaces that are pre-inoculated with a known microbial consortium. Film media are only recently being assessed for cultivable microbial counts in extremely low-biomass environments such as clean rooms and hospitals [31,32,33]. Recently, researchers from NASA compared Petrifilm Rapid Aerobic Count (RAC) plates with tryptic soy agar (TSA) plates for measuring the bioburden of an extremely low-biomass clean room environment and found that Petrifilm was equal to TSA plates in aerobic bacterial estimation [31]. In addition, Petrifilm was found to be superior to TSA in containing colonies that could spread and grow to cover 25% or more area of agar plate [31].

Kikkoman’s Easy Plates have not yet been compared in the scientific literature for their performance relative to both agar plates and Petrifilm. While film media have been previously used in place of agar plating to assess cultivable microbial counts for built environments and hospitals [30], they have not been tested in extremely low-biomass environments, particularly those with high fiber counts. Additionally, because Easy Plates entirely rely on water adhesion/cohesion forces for inoculation rather than manual spreading, it is likely that they could be viable options for environmental monitoring in micro- or low-gravity settings in future space installations.

Therefore, our objectives for this study were to (1) test the bacterial enumeration performance among agar plates, Petrifilm, and Easy Plates for a variety of food items; (2) compare the cultivable microbial counts in an Earth-built-environment setting for agar plates, Petrifilm, and Easy Plates using bacterial- and fungal-specific media; and (3) test the efficacy of Easy Plates as an alternative method for bacterial and fungal bioburden estimation and the isolation of bacterial and fungal strains for surface samples from the ISS, for both cultivable microbial counts and species composition of isolates. 

## 2. Materials and Methods

### 2.1. Descriptions of Petrifilm and Easy Plate Media Tested

According to the manufacturer’s instruction manuals, Petrifilm Aerobic Count (AC) Plate [15] (3M, Saint Paul, MN, USA, Cat# 6400) and Easy Plate AC [16] (Kikkoman, Noda, Chiba, Japan, Cat# 61973) were used for the enumeration of aerobic bacteria, and Petrifilm Rapid Yeast and Mold Count (RYM) Plate (3M, Cat# 6475) and Easy Plate YM-R (Kikkoman, Cat# 61977) were used for the enumeration of yeast and mold in the food and beverage industries. In general, in food and beverage microbiological testing, both the Petrifilm AC and Easy Plate AC count red colonies after 48 h of incubation at 35 °C. Petrifilm RYM counts blue-green colonies after 48 h of incubation at 25 °C or 28 °C, and Easy Plate YM-R counts purple colonies after 48 h at 25 °C. Both media can extend the incubation time up to 3–7 days if the colonies appear faint.

### 2.2. Food-Item Cultivable Microbial Counts and Media Testing

Food items (n = 63), including raw and cooked meats, vegetables, nuts, and processed foods (see Supplementary Table S1 for list), were homogenized with 90 mL (10 g samples) or 225 mL (25 g samples) of sterile phosphate-buffered saline (PBS), and 10-fold serial dilutions were performed. One milliliter of each dilution was inoculated into each medium (Plate Count Agar, Petrifilm AC, and Easy Plate AC), incubated for 48 h at 35 °C, which are standard FDA methods for food safety testing [34], and the number of bacteria was counted. Fungal counts were not assayed for food samples.

### 2.3. Built-Environment Cultivable Microbial Counts and Media Testing

The surfaces of Japanese homes (n = 5) were sampled for bacterial and fungal cultivable microbial counts. At each home, three different surfaces were sampled: one expected low-bioburden (example: window glass), one expected medium-bioburden, and one expected high-bioburden (example: living room floor) sample. Pro-media ST-25 phosphate-buffered saline (PBS) swab kits from ELMEX LIMITED (Shinjuku-ku, Tokyo, Japan), consisting of a cotton swab in 10 mL of sterile PBS, were used to swab the surfaces and were kept refrigerated (~4 °C) until use.

In each home, for each surface, a 100 cm^2^ area was sampled in all cases except for complex shapes (such as doorknobs) in which case the shape was thoroughly wiped over its entire surface area, and the swab was returned to the sterile PBS and shaken. Aliquots of 1 mL of undiluted and 1 mL of 10^−1^ diluted samples were plated on 6 types of media for each sample: traditional plate count agar (PCA) pour plate, Easy Plate AC, and Petrifilm AC to target aerobic bacteria, and conventional potato dextrose agar (PDA, BD Diagnostics Cat #213400) with 0.05 g/L chloramphenicol pour plate, Easy Plate YM-R, and Petrifilm RYM to target environmental fungi. Plates were incubated for 7 days at 25 °C, with cultivable microbial counts taken on Day 2 and Day 7; however, if plates were close to being too numerous to count on Day 2, colonies were counted and not incubated further. 

### 2.4. International Space Station Sampling and Media Testing

*Preparation of inoculum:* Methods for preparing sampling kits and processing samples were similar to those used in the Microbial Tracking 1 and 2 missions [3,35,36]. Sterile TexTra™ 10 TX3224 Dry Cleanroom Wipers were moistened with 15 mL of sterile phosphate-buffered saline (PBS), folded into quarters, and placed in sterile Ziploc baggies, which were later used to assemble the sampling kits at NASA Ames. Sample kits were transported to the ISS in June 2021. Over the course of five days, astronauts sampled the same square meter area of the same eight surfaces each day (see [3] for detailed surface descriptions). On each sampling day, for each surface, the astronaut tasked with sampling donned a new pair of sterile gloves, removed and unfolded a sterile wipe from its bag, and wiped a square meter area of the surface three times, folding the wipe in half for the second pass and into quarters for the third pass. Additionally, on each sampling day, a wipe was removed from its bag and briefly exposed to the air, as an environmental control sample. A total of 45 samples (8 surfaces + 1 control, ×5 days) were produced and stored at 4 °C aboard the ISS and during transport to Earth.

Upon delivery to the Jet Propulsion Laboratory (JPL), the wipes were separated into batches and stored at 4 °C until processing. All wipes were processed within 48 h of arrival at the JPL for the isolation of environmental microbes. All lab procedures were performed for all samples within a 12 h period. Each wipe was placed in a sterile 500 mL bottle with 200 mL sterile PBS and vigorously shaken for 2 min. In addition to the 45 ISS samples, an unopened wipe and a bottle of a sterile buffer without the addition of wipes were used as negative controls; no colonies resulted from these samples; thus, those data are not reported here. The PBS was then concentrated using an InnovaPrep CP-150 (Innovaprep LLC, Drexel, MI, USA) with a 0.2 µm Polysulfone hollow-fiber-concentrating pipette tip. Using additional sterile PBS, the resulting PBS concentrate was adjusted to 4 mL for all samples, and dilutions of 10^−1^, 10^−2,^ and 10^−3^ were prepared. 

*Inoculation and cultivable microbial counting:* Two replicate spread plates were prepared for all three dilution levels (10^−1^, 10^−2,^ and 10^−3^), using two types of agar media: Reasoner’s 2 agar (R2A, BD Diagnostics Cat # 218263) and PDA plates supplemented with 0.05 g/L chloramphenicol, for a total of 12 plates per sample (2 agar types × 3 dilution levels × 2 replicates; ~540 plates in total). This occupied a total of almost 0.1 cubic meters, the equivalent of a large moving box. For each of the two types of film media (Kikkoman Easy Plate AC and Easy Plate YM-R), 1 mL of the 10^−1^ dilution was directly pipetted into the center of each plate and then sealed according to the manufacturer’s instructions. Agar and film plates were incubated at room temperature for 7 days; cultivable microbial counts were performed on Days 2 and 7. For ISS samples, PetriFilm media were not used.

*Isolation, purification, and identification of ISS strains:* After cultivable microbial counting, up to 5 isolates were chosen from each combination of media type and sample. On both the agar plates and Easy Plates, colonies were deliberately selected for morphological differences, size, and colony coloration on agar plates. Colonies from Easy Plates were first streaked out onto TSA/PDA plates, incubated at 25 °C for 7 days, and imaged, before being transferred to 1:10 agar stabs of respective media. Similarly, the colonies from agar plates were directly transferred to respective agar stabs. The isolates were later streaked out for purification onto TSA or PDA plates. The secondary streak plates of purified isolates were sent to Azenta Life Sciences (Southfield, NJ, USA) for colony PCR + Sanger sequencing for the 16S rRNA gene or the ribosomal Internal Transcriber Space (ITS) region, respectively, for bacteria and fungi. Primers 27F and 1429R (27F 5′-AGAGTTTGATCCTGGCTCAG-3′; 1492R 5′-GGTTACCTTGTTACGACTT-3′) [37] were used to target the entire 16S region (V1-V9) for bacterial strains. ITS regions 1 and 2 were targeted for fungal taxonomy using primers ITS1-F and ITS4 (ITS1 5′-CTTGGTCATTTAGAGGAAGTAA-3′; ITS4 5′-TCCTCCGCTTATTGATATGC-3′) [38]. Per Azenta Life Sciences standard operating procedure, enzymatic cleanup was performed after amplification using Exonuclease I—Shrimp Alkaline Phosphatase (ExoSAP), and dye-terminator Sanger sequencing was performed using an Applied Biosystems BigDye version 3.1 sequencing kit using an Applied Biosystem 3730xl DNA Analyzer.

*Statistical analysis:* All statistical analyses and figure creation were carried out in R. Consensus 16S rRNA gene and ITS sequences were generated using the sangeranalyzeR package [39], and genus/species identity was determined using the nearest match to the NCBI nucleotide database. Type II linear models, Spearman and Pearson’s correlation coefficients, and paired and unpaired Wilcoxon rank-sum tests were used to assess the correlations and mean differences between the cultivable microbial counts of agar and film media. Although a 1 m^2^ area was used for ISS samples, for this research communication, we have converted the unit to cultivable microbes per 100 cm^2^ in order to illustrate the extremely low bioburden of the ISS samples compared with that of the food-item and built-environment studies also sampled.

## 3. Results

**Figure 1:**
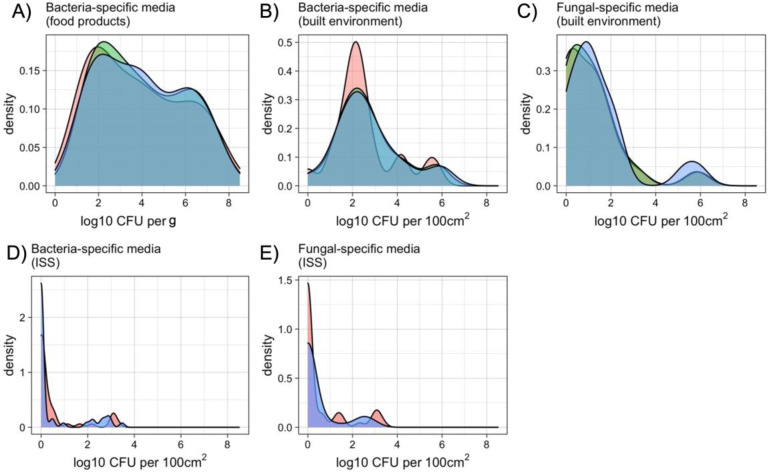
Density distribution for overall food items (**A**), Earth-built-environment (**B**,**C**), and ISS surface (**D**,**E**) cultivable microbial counts for all media types. In food-item and Earth-built-environment testing (**A**–**C**), agar was compared with both Petrifilm and Easy Plates, while in ISS surface sampling (**D**,**E**), agar was only compared with Easy Plates.

The microbial population enumerated with traditional agar plate, Petrifilm, and Easy Plate are depicted in Figure 1. All three types of media were used for the comparison of food products (Figure 1A) and built environments (Figure 1B for bacteria and Figure 1C for fungi). Only the R2A medium was compared with the Easy Plate method for estimating the bacterial population (Figure 1D) of the ISS samples. Similarly, the PDA medium and Easy Plate were employed to enumerate the fungal population (Figure 1E) from the ISS environmental surfaces. 

### 3.1. Cultivable Population of Food Items

**Figure 2:**
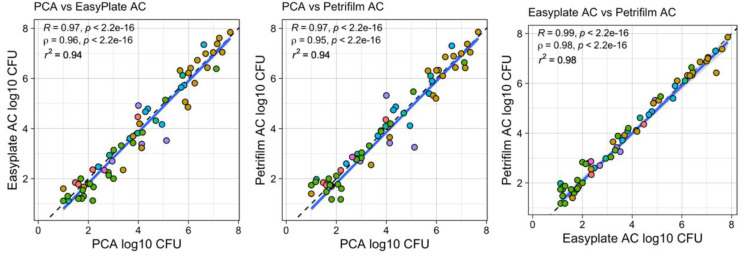
Correlation of agar and film media counts for PCA vs. Easy Plate AC, PCA vs. Petrifilm AC, and Easy Plate AC vs. Petrifilm AC for a variety of food items. Cultivable microbial counts are per milliliter, from swabbing food items and placing the swab in a 10 mL buffer solution. Solid blue line shows linear regression.

Aerobic bacterial counts for food items ranged from 10^2^ to 10^9^ cultivable microbes per gram (Figure 1A). The counts correlated well between media types, with R^2^ values of 0.94 or greater and no significant difference between the mean bacterial cultivable microbial counts for each media type (Table 1, Figure 2). The mean log10 differences between media types were <2.5 (i.e., less than 10^0.34^) colonies in all cases. Paired Wilcoxon tests between film media and agar were not significant but were significant between Easy Plate and Petrifilm media, with Easy Plate having slightly higher cultivable microbial counts. When broken down by food category, only the microbial community of raw poultry did not show a high correlation between agar and film media counts. However, the correlation between Petrifilm and Easy Plate cultivable microbial counts was high for all food categories (Table 2).

### 3.2. Cultivable Population of Built-Environment Surfaces 

**Figure 3:**
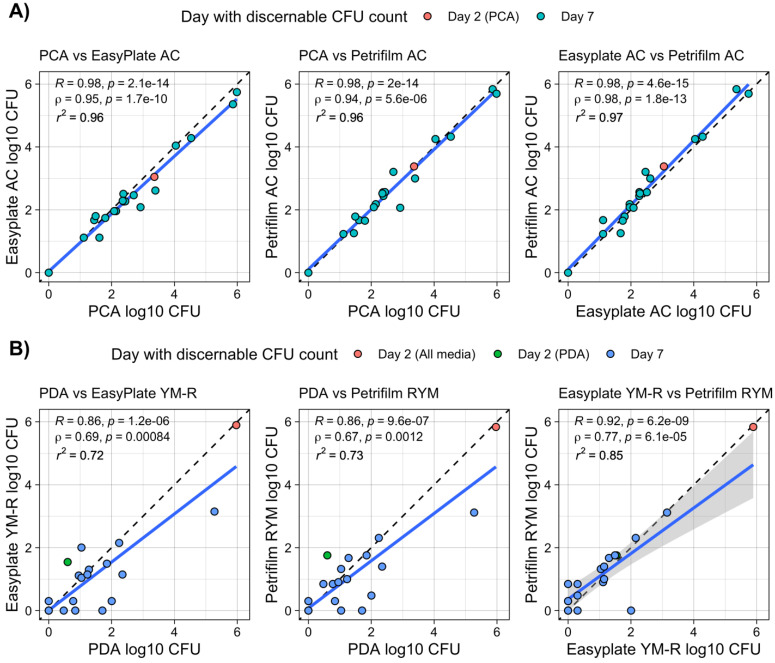
Cultivable microbial counts for built-environment surfaces in households in Japan for (**A**) bacterial-specific and (**B**) fungal-specific media, compared between film and agar media and media specificity type. PDA = potato dextrose agar; PCA = plate count agar. All cultivable microbial counts are per 100 cm^2^ of surface area. Blue solid line is linear regression; shading is 95% confidence interval.

Cultivable microbial counts for both bacterial- and fungal-specific media ranged from below the detection limit to 10^6^ per swab (per 100 cm^2^ surface area) for built-environment surface samples (Figure 1B,C). There were no significant differences between mean cultivable microbial counts among the three media types, for either bacterial- or fungal-targeted media. In all cases, mean log10 differences between media types were less than 2.5 colonies (i.e., <10^0.34^). Similar to the results for food items, the correlations between bacterial-specific film and agar media were high, with R^2^ values between 0.96 and 0.97, and no significant differences between the mean cultivable microbial counts were detected (Table 1, Figure 3). The correlations between cultivable microbial counts for fungal-specific media were lower than those for bacteria, with R^2^ values of ~0.73 for both Petrifilm RYM and Easy Plate YM-R when correlated to PDA. Easy Plate YM-R and Petrifilm RYM had higher correlations between them (R^2^ = 0.85), but there was still considerable variation in their counts. Paired Wilcoxon tests showed that Easy Plate AC had slightly lower cultivable microbial counts than both PCA and Petrifilm AC, which, as stated previously, was not large enough to cause a difference in mean cultivable microbial counts. The mean log10 differences between the media types were <0.34 (i.e., a non-log difference of 2 or fewer colonies) in all cases.

### 3.3. Cultivable Population of the International Space Station 

**Figure 4:**
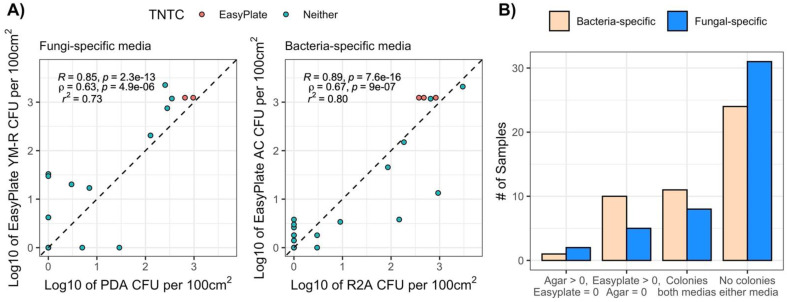
(**A**) Correlations between EasyPlate vs. agar cultivable microbial counts for fungal-specific (PDA vs. EasyPlate YM-R) and bacterial-specific (R2A vs. Easy Plate AC) media for ISS samples. TNTC = too numerous to count. TNTCs are included as arbitrarily high numbers in this figure but are not included in model calculations; (**B**) number of samples with zero vs. non-zero values for agar media vs. Easy Plates for ISS samples.

Cultivable microbial counts ranged from below the detection limit to 10^3^ per wipe for both bacterial- (Figure 1D) and fungal-specific (Figure 1E) media (per 100 cm^2^ surface area). There was no difference between the mean cultivable microbial counts for Easy Plate AC vs. R2A, nor for Easy Plate YM-R vs. PDA (Table 1). The cultivable microbial count correlations were similar in food and Earth built environments for fungi, with R^2^ values of 0.73 for fungal-specific media, but were lower than Earth-based studies for bacteria-specific media, with an R^2^ of 0.80 (Figure 4A). This was due in part to the number of samples that had zero colonies for one or both media types (Figure 4B). The samples plated on Easy Plates, both AC and YM, were more likely to have cultivable microbial counts above the detection limit compared with agar media (Figure 4B).

### 3.4. Specificity of Media Types for Bacteria or Fungi

The differential isolation of bacteria and fungi by traditional agar and Easy Plate methods is shown in Figure S1. Among the 40 samples tested, 10 samples yielded at least one bacterial colony in Easy Plate AC but were not present in the R2A medium. In contrast, one R2A medium showed growth, whereas the Easy Plate AC method failed to yield any colony. The further isolation, purification, and identification of the bacteria grown in the R2A plate revealed the presence of *Sphingomonas lutea*. The pure strain of *S. lutea* isolate was regrown in the Easy Plate AC medium and showed growth. Similarly, among the 40 samples tested for fungal population estimation, 6 samples showed growth of at least one fungal colony in Easy Plate YM-R but did not show growth in the PDA medium. In contrast, two PDA plates showed growth, whereas the Easy Plate YM-R method failed to yield any colony. Further ITS-based identification using blast searches against the NCBI database revealed that these fungal isolates were closely related to *Aureobasidium leucospermi* and *Penicillium rubens*, and subsequent growth in Easy Plate YM-R yielded growth, revealing that there was no inhibition in growth in the Easy Plate YM-R medium.

**Figure 5:**
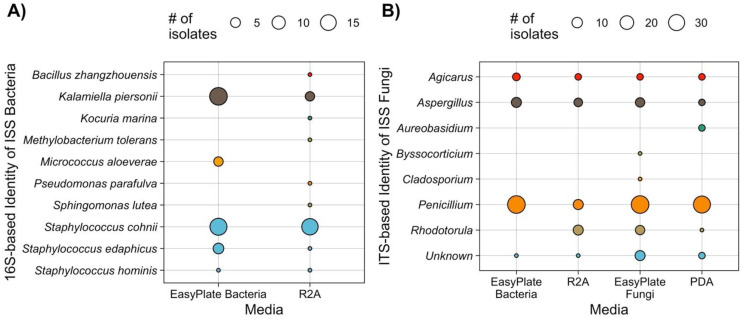
(**A**) Distribution of bacterial species isolated from Easy Plate AC vs. R2A agar media; (**B**) distribution of fungal genera isolated from Easy Plate AC and YT, and R2A, and PDA agar media. Points are colored by genus.

For ISS samples, neither PDA + chloramphenicol agar media nor Easy Plate YM-R (which also contains chloramphenicol) isolated bacterial strains, but R2A and Easy Plate AC both promoted the growth of a significant number of fungi (Figure 5B).

### 3.5. Species and Genus Distributions for Bacterial and Fungal Isolates

The dominant bacterial species isolated from Easy Plate AC and R2A were similar, with multiple strains of *Staphylococcus* species and *Kalamiella piersonii* isolated by both types of media (Figure 5A). However, after 7 days of growth, a single isolate of five slow-growing species was isolated from R2A (*Methylobacterium tolerans, Kocuria marina, Bacillus zhangzouensis, Psueodomonas parafulva*, and *Sphingomonas lutea*) but not from Easy Plate AC, while multiple strains of *Micrococcus aloverae* were isolated from Easy Plate AC but not from R2A. In addition, the16S rRNA gene-sequence-based species identity was at 97% or above for all the isolates and above 99% for most. Since the ITS is not suitable to define fungal species, the genus-level identification was recorded. The fungal identification for isolates (Figure 5B) was similar across the board, with multiple strains of *Penicillium, Aspergillus*, and *Agicarus* isolated from R2A, PDA, and Easy Plate AC and YM-R. Easy Plate YM-R also isolated a single strain of *Cladosporium* and *Byssocorticium*, while PDA isolated a single strain of *Aureobasidium*. A more significant difference was the lack of the yeast genus *Rhodotorula* from Easy Plate AC isolates, whereas R2A plates promoted the growth of *Rhodotorula* species. We, therefore, plated a *Rhodotorula* isolate onto Easy Plate AC while also inoculating Easy Plate YM-R, R2A, and PDA with the same cell dilution for comparison. While *Rhodotorula* grew on Easy Plate AC medium, it was simply not visible to the eye, and the only way to detect it was to plate out random locations from the Easy Plate on different media. This was in contrast to non-yeast fungal species, which were highly visible on Easy Plate AC.

## 4. Discussion

In general, the use of film media over agar allows for lower costs, greatly increased inoculation speed, greatly reduced experimental preparation time, and much smaller amounts of space required in incubators or counters; these advantages have been discussed in numerous previous reports [28]. When dealing with high volumes of samples that must be processed in a very short amount of time, film media such as Petrifilm and Easy Plates provide distinct advantages. In processing the ISS samples, plating the same number of samples on Easy Plates took one person 2 h, as opposed to three people taking 8 h to inoculate the agar plates. The use of film media or pour plates requires fewer dilutions than spread plates because it can produce accurate plate counts at higher bioburden levels, which is not necessarily a fair comparison of the time spent. However, in addition to a pipetting step, spread plates require either retrieving a pre-sterilized spreader from its packaging or sterilizing a reusable spreader and thoroughly spreading the sample over the surface without damaging the agar. For upwards of 300 agar plates per experiment, this adds up to multiple hours. 

In this study, we found no particular advantage or disadvantage to using Petrifilm (3M) vs. Easy Plates (Kikkoman), except for the lack of necessity of using a spreader for Kikkoman plates. The cultivable microbial counts for built-environment samples and food samples correlated extremely well with counts from agar media and with each other. The one exception to this trend was the bacterial counts from raw poultry products, where Petrifilm and Easy Plate cultivable microbial counts correlated well with each other but did not correlate with agar; given the small number of poultry samples (n = 4), this trend would need to be investigated further to be considered significant.

The ranges of cultivable microbial counts for built-environment samples and ISS samples in this study were considerably lower, including many samples below the detection limit for one or both types of media, than almost all the previously published studies using film media in other environments. Most ISS samples had low cultivable microbial counts that were below the cultivable microbial count standard for hospital cleanliness (2.5 × 10^2^ cultivable microbes per 100 cm^2^, OR 10^2.4^ per 100 cm^2^) [32]. The correlations between cultivable microbial counts for fungal-specific agar and film media were lower than the correlations for bacteria and ranged from adjusted R^2^ values 0.73 to 0.82 for built-environment samples and ISS samples. The correlation between bacterial-specific agar vs. film media was lower for ISS samples (adj R^2^ = 0.80) than Earth built environments (R^2^ = 0.94–0.98), probably due to both the low bioburden in ISS samples and the presence of considerable fungi growing on bacterial-specific media, which might have masked the presence of bacteria. It is noteworthy to mention here that the increase in inoculum size (1 mL) yielded cultivable counts more in film media (10 samples for bacteria and 6 samples for fungi; Figure 5) than the traditional agar(s) used (100 µL).

The species distribution for film media vs. agar media was similar between both fungal-specific and bacteria-specific plates. Three main differences were that (1) yeast colonies grew but were not visible on Easy Plate AC (although non-yeast fungal colonies were visible and countable), whereas yeast colonies were of course visible on R2A; (2) Easy Plates isolated multiple strains of *Micrococcus*, whereas R2A did not; and (3) isolation from R2A allowed for the isolation of single strains of slow-growing low-nutrient-loving bacterial species not isolated on Easy Plates, from minuscule colonies. In some cases, the minuscule colony had a distinct color that allowed us to select it as a colony of interest from the agar, whereas on the Easy Plate, it was not distinguishable from other colonies; in some cases, there was only a single tiny colony on the agar plate vs. no colony on the Easy Plate, which could be due to random chance: In at least two cases, there would have been no way to pick the rare species out, and it was chance that led us to collect it. Inoculating well-grown colonies isolated from R2A onto Easy Plate AC medium allowed the growth of these minuscule colonies. Picking colonies out by their relative size on film media is difficult, as the dye used in Petrifilm and Easy Plates magnifies the size of tiny colonies. Additionally, without a clear view of colony morphology and color that agar provides, it is possible that isolating “rare” species might have been overlooked. This could be an advantage or disadvantage of film media depending on the goals and study design (it removes non-random sampling biases, but if one is sampling non-randomly, it may result in a lower diversity of isolates).

Other disadvantages of film media are that (1) because of the smaller surface area and the miniaturization of colonies, it was difficult to count colonies by eye for high bioburden samples, and in a few cases, counting from magnified photographs was necessary (3M has developed an automated CFU counter for use with Petrifilm plates, which negates this issue), and (2) because colony color was not observable, and morphology was somewhat obscured in film media, it would be extremely difficult to rule out the colonies of a known contaminant that appeared also in control plates (for example, in a situation where samples are plated out in the field or in otherwise non-laboratory conditions).

Surfaces in built environments inhabited by humans and animals are inherently difficult to consistently sample for cultivable microbial counts, due to having higher fungal:bacterial ratios and the presence of hairs and fibers, which adsorb bacterial and fungal spores, thus requiring multiple replicates to obtain an accurate count. Even with every effort made to vortex or otherwise homogenize samples before plating, in extremely low-biomass environments, the presence or absence of a single hair or fiber might make the difference between a zero and non-zero cultivable microbial count, or a lower or higher cultivable microbial count. Human habitation and the accompanying presence of dust, fibers, and hairs from bodies and cloth, combined with extremely low surface biomass due to constant cleaning and well-filtered air, is a combination peculiar to hospital environments [40], clean rooms [41], and space habitats [42,43]; however, because astronauts do not wear masks, gloves, or other protective equipment aboard the ISS, the problem of hairs and fibers is exacerbated. One study tested the efficacy of Petrifilm vs. contact plates on regular hospital surfaces and found that Petrifilm detected larger numbers of cultivable microbes [32]; however, cultivable microbial counts in that study were higher than what would be considered “low bioburden” (i.e., comparable to a clean room or the ISS). In a study of debris and lint collected via vacuuming the ISS surfaces, fibers and hairs—as opposed to tinier particles such as skin cells with a greater surface area—yielded a distinct microbial community with greater fungal diversity and only one order of magnitude fewer cultivable microbes than powdery debris despite a vastly smaller surface area [42]. Therefore, the use of a larger volume is critical for accurate plate counts. As film media use a 10× higher volume than traditional spread plates, while not presenting the same challenges of possible heat death (molten agar at 55 °C) and excavation that isolating bacteria and fungi presents when using pour plates [44,45], film media are well-suited for use with samples from the ISS and other future space habitats. In fact, because film media are extremely light, and thin, and rely on water cohesion and adhesion rather than gravity or manual spreading, they may be suitable for testing in microgravity in the future and may be a possible method for both food and environmental safety testing for future space habitats.

## Figures and Tables

**Table 1:** Results of paired and unpaired Wilcoxon tests and type II linear models comparing between media types. Bacterial-specific agar media were PCA for food items and built environment; R2A for ISS samples. Fungal-specific agar media were PDA w/chloramphenicol for all studies.

				Wilcoxon		Type II Regression (MA)			
Media Target	Study	X	Y	Paired ^a^	Unpaired	Intercept	Slope	R2	Mean log10 Difference
Bacteria-specific	Food items	Agar	Easy Plate		--	–0.36	1.05	0.94	−0.143
	Food items	Agar	Petrifilm	--	--	0.01	0.99	0.94	−0.005
	Food items	Easy Plate	Petrifilm	**	--	0.34	0.95	0.98	0.138
	Built Environment	Agar	Easy Plate	**	--	−0.002	0.93	0.96	−0.186
	Built Environment	Agar	Petrifilm	--	--	0.06	0.97	0.96	−0.018
	Built Environment	Easy Plate	Petrifilm	**	--	0.07	1.04	0.97	0.169
	ISS	Agar	Easy Plate	--	--	0.06	0.74	0.80	−0.044
Fungal-specific	Built Environment	Agar	Easy Plate	--	--	0.01	0.77	0.72	−0.338
	Built Environment	Agar	Petrifilm	--	--	0.05	0.75	0.73	−0.29
	Built Environment	Easy Plate	Petrifilm	--	--	0.16	0.9	0.85	0.048
	ISS	Agar	Easy Plate	*	--	0.12	1.06	0.73	−0.14

^a^. * corresponds to *p* < 0.05; ** corresponds to *p* < 0.01

**Table 2:** Paired Wilcoxon test results (P adjusted for multiple comparisons using FDR correction) and Pearson’s rho for bacterial counts for food samples, by food category.

		Pearson’s Rho	
Food Category	n	Easy Plate AC: PCA	Petrifilm AC: PCA
Multi-component foods or meal Components	21	0.97	0.96
Fresh produces and fruits	20	0.96	0.97
Raw meat and ready-to-cook meat products (except poultry)	8	0.95	0.94
Raw and ready-to-cook fish and seafood (unprocessed)	5	0.99	0.99
Raw poultry and ready-to-cook poultry products	4	0.33	0.17
Dried cereals, fruits, nuts, seeds, and vegetables	4	1	1

## Data Availability

Raw CFU data are available under Supplementary Table S1 (food items), Table S2 (built environment). ISS data will be released upon publication of initial results for the Microbial Tracking 3 project.

## Supplementary Materials

The following supporting information can be downloaded at: https://www.mdpi.com/article/10.3390/microorganisms10091714/s1, Figure S1: Morphology of strains captured by film media but not agar media or vice versa; Table S1: CFU count data for food study; Table S2: CFU count data for built environment, Japan study.
