# Supplementary material for: Microbial Burden Estimation of Food Items, Built Environments, and the International Space Station Using Film Media
Source: Microorganisms. 2022 Aug 25;10(9):1714. doi: 10.3390/microorganisms10091714 (PMC9503880; doi:10.3390/microorganisms10091714)
Supplement: Supplementary file 1 [file microorganisms-10-01714-s001.zip › Figure S1.pdf]

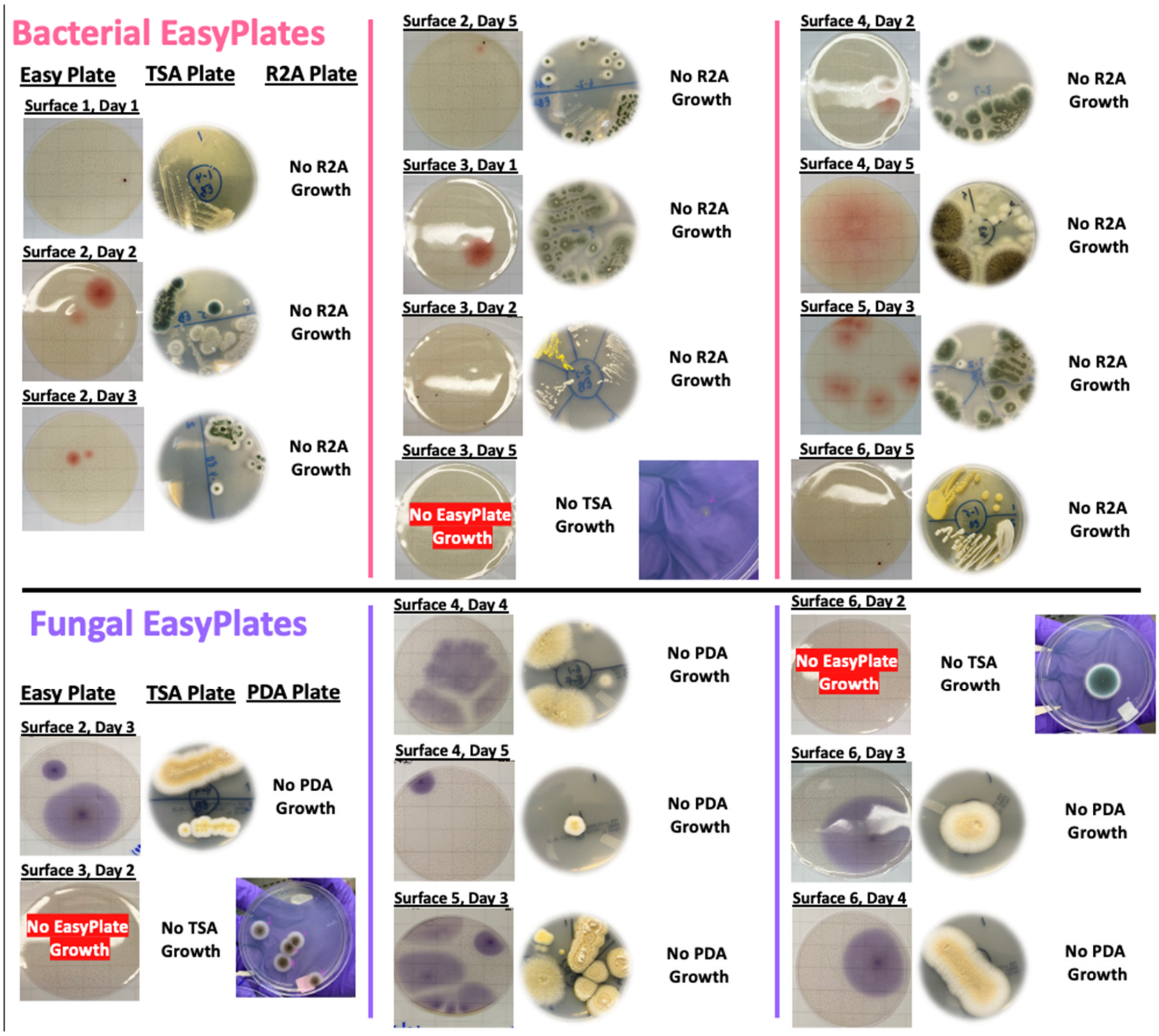


Figure S1: Morphology of strains captured by film media but not agar media or vice versa
